# Characteristics of cognitive impairment in adult asymptomatic moyamoya disease

**DOI:** 10.1186/s12883-020-01898-8

**Published:** 2020-08-31

**Authors:** Shihao He, Ran Duan, Ziqi Liu, Xun Ye, Li Yuan, Tian Li, Cunxin Tan, Junshi Shao, Shusen Qin, Rong Wang

**Affiliations:** 1 Department of Neurosurgery, Beijing Tiantan Hospital, Capital Medical University, 119 South Fourth Ring West Road, Fengtai District, Beijing, 100070 China; 2 Department of Neurosurgery, Peking University International Hospital, Beijing, China; 3 State Key Laboratory of Cognitive Neuroscience and Learning & IDG/Mc Govern Institute for Brain Research, Beijing Normal University, Beijing, China; 4 Center of Stroke, Beijing Institute for Brain Disorders, Beijing, China

**Keywords:** Moyamoya disease, Cognition impairment, Asymptomatic

## Abstract

**Background:**

Cognitive impairment in adult moyamoya disease (MMD) is thought to be the result of ischemic stroke; however, the presence and extent of cognitive decline in asymptomatic patients is unclear.

**Methods:**

After classification using T2-weighted fluid attenuated inversion recovery (FLAIR) magnetic resonance imaging (MRI), a total of 19 MMD patients with a history of cerebral infarction, 21 asymptomatic MMD patients, and 20 healthy controls matched for age, sex, and years of education were prospectively included in this study. A detailed neuropsychological evaluation of two moyamoya subgroups and normal controls was conducted.

**Results:**

Asymptomatic patients showed varying degrees of decline in intelligence (Raven’s Standard Progressive Matrices, *P* = 0.001), spatial imagination (mental rotation, *P* = 0.014), working memory (verbal working memory-backward digit span, *P* = 0.011), and computational ability (simple subtraction, *P* = 0.014; complex subtraction, *P* < 0.001) compared with normal controls. MMD patients with cerebral infarction had more severe impairment in complex arithmetic (*P* = 0.027) and word short-term memory (*P* = 0.01) than those without symptoms.

**Conclusion:**

In asymptomatic MMD patients, a variety of cognitive impairment precedes the onset of clinical symptoms such as cerebral infarction, which may be a long-term complication of conservative treatment.

## Background

Moyamoya disease (MMD) is an uncommon cerebrovascular disease with relatively high incidence in East Asia, especially in Japan, China, and South Korea [1]. MMD is a cerebrovascular disease of unknown etiology characterized by chronic progressive stenosis or occlusion of the distal bilateral internal carotid artery and the presence of unusual reticular vessels in the basal part of brain [2]. In addition to being more prone to cerebrovascular accidents, such as ischemic cerebral infarction and cerebral hemorrhage, patients with MMD may exhibit cognitive impairment [3, 4]. At present, there is no curative treatment for moyamoya disease. Secondary prevention for patients with symptomatic MMD is largely centered on surgical revascularization techniques [5].

Many studies have suggested that stroke is an important cause of cognitive and affective disorders in patients [6, 7]. Even in China, the epidemiology of asymptomatic MMD remains obscure and guidelines for its treatment have not yet been established [8–10]. In the recent years, the development of non-invasive diagnostic methods, including MRI and MRA, and the emphasis on physical examination may lead to a higher incidence of asymptomatic MMD than previously thought. “Asymptomatic” MMD patients were previously defined as those who had neither an hemorrhage attack nor an ischemic attack [11, 12]. However, cognitive impairment not only occurs in symptomatic MMD patients with a history of cerebral infarction but also in asymptomatic MMD patients. An experimental study of a chronic cerebral ischemia model in adult mice showed that although the mice still maintained spontaneous activity during hemodynamic disorder caused by chronic cerebral instillation, they showed an obvious object recognition disorder [13]. A cognitive study of MMD patients with no history of cerebral infarction found that executive function, mental efficiency, and word finding were the most frequent impaired abilities, whereas memory was relatively intact [14]. These studies suggest that asymptomatic MMD is not necessarily “asymptomatic” but that the advanced functions of the brain have not been evaluated well by previous studies. Therefore, it is necessary to clarify the cognitive function in asymptomatic patients in order to develop guidelines for their treatment.

MMD patients have moderate to severe impairment in different cognitive domains. However, the reported incidence and severity of impairment varies from study to study [15]. In particular, a subgroup analysis was not carried out in many studies and the cognitive tests used were mostly simple cognitive screening scales. At the same time, no study explored the cognitive similarities and differences between patients with a history of ischemic stroke and asymptomatic MMD patients to understand the initial stages and causes of cognitive impairment in MMD patients.

Therefore, the purpose of this study was to investigate the differences and similarities in cognitive impairment in different subgroups of MMD patients using comprehensive cognitive tests. This study provided cognitive test results and statistical evidence of differences in intelligence, spatial imagination, number operation, short-term memory, and executive function between different MMD patient subgroups and healthy controls.

## Methods

### Participants

This prospective study enrolled 40 patients with MMD at the Neurosurgery Department of Beijing Tiantan Hospital affiliated to Capital Medical University between June 2019 and April 2020. We also recruited 20 age, sex, and education level-matched healthy controls in this study.

**Clinical Trial Registration-URL**: http://www.chictr.org.cn.

Unique identifier: ChiCTR1900023610. Registered 4 June 2019 – Prospective study registered.

### Inclusion and exclusion criteria

The inclusion criteria were as follows:

(1) All patients should meet the Guidelines for Diagnosis and Treatment of Moyamoya Disease (Spontaneous Occlusion of the Circle of Willis), the research committee on the pathology and treatment of spontaneous occlusion of the circle of Willis, and health labor sciences research grant for research on measures for intractable diseases [16]; (2) for inclusion in the cerebral infarction group, an infarction that is 1.6–5.0 cm in diameter on T2 MRI for the infarct lesions corresponding to the patient’s symptoms; (3) for inclusion in the asymptomatic MMD patients group, no previous history of ischemic or hemorrhagic attack, and in the case of intracranial lacunar cerebral infarctions, the lesion diameter should be less than 1.5 cm (4) right-handed dominance; and (5) no major psychiatric disease, such as dementia or depression, or other medical condition.

The exclusion criteria were as follows: (1) acute stage of cerebral infarction and other neuropsychiatric diseases, severe systemic diseases, and severe systemic diseases (e.g., Alzheimer’s disease, Parkinson’s disease); (2) any contraindications for MR scans (e.g., metal implants); (3) use of any medications that could affect cognitive abilities; (4) fatigue or hunger; or (5) an inability to complete the tasks independently.

### Neuropsychological assessments

All cognitive assessments were performed using the Online Psychological Experimental System. Each task had two sessions, a practice session and formal testing session. All tasks have shown acceptable half-split reliabilities, ranging from 0.80 to 0.96 according to previous studies [17–19].

Nonverbal matrix reasoning was used to assess general intelligence and abstract reasoning ability, which has been correlated with mathematical performance [20, 21]. The task was adapted from Raven’s Standard Progressive Matrices (Raven, 2000) [22]. Each question had four to six potential responses and only one was the correct answer. Participants were asked to identify the missing image from a sequence according to the rules behind it.

Mental rotation was used to evaluate visuospatial ability. The test was adapted from the study by Vandenberg and Kuse (1978) [23]. The revised version had only two choices and was limited to 3 min. There were three 3D images in each trial, one shown at the top of the screen and the other two at the bottom. Participants were asked to judge which of the two candidates at the bottom was the same as the top one, after mentally rotating one of the images. The correct image was rotated from the original, with a rotation angle ranging from 15° to 345° (at intervals of 15°). The other image was a mirror image of the target. Participants pressed the “Q” key to select the image on the left side or “P” to select the image on the right side. An adjusted number of correct trials was used (see the sentence completion test).

A verbal working memory (VWM) test was used to measure working memory capacity. The digit span test from the Wechsler intelligence scale was used. The test was divided into two parts, a forward digit span task representing short-term memory and general attention and a backward digit span task representing working memory related to executive function [24, 25].

Simple subtraction was used to assess simple calculation ability and magnitude representation [19]. The task involved 92 subtraction problems with a correct single-digit answer. For each trial, a subtraction question (for example, 8–3) appeared at the top of the screen, and two candidate answers appeared at the bottom. The minuend of each question ranged from 2 to 18 and answers ranged from 2 to 9. The official test time limit was 4 min.

In the complex subtraction task, there were 95 problems, with each problem involving double-digit numbers for both operands. Most problems required Mathematical borrowing. In each trial, a subtraction problem (e.g., 63–27) was presented at the top of the screen, with two candidate answers presented at the bottom. The difference between the true and false answers was either 1 or 10. Formal testing of the task was limited to 3 min.

Word-memory ability and visual short-term memory were measured using the short-term memory span for Chinese words and phrases and the picture short-term memory test, respectively [26]. During the learning stage, a series of words and pictures were presented on the screen in turn. In the test, the subjects judged whether the test images had been presented in the learning phase. If so, they pressed the “Q” key using their left index finger; otherwise, they pressed the “P” key with their right index finger.

The Edinburgh Handedness Inventory was used to investigate left and right-handedness.^18^ The subjects filled in the Edinburgh handedness questionnaire and a final score ≥ 4 was classified as right-handedness, while scores ≤ − 4 were classified as left-handedness; intermediate scores were classified as double handedness [27].

The participants were tested using computer workstations by neuropsychologists, who were blinded to the patient’s clinical data. The interval between neuropsychological testing and MRI examination was < 5 days.

### Statistical analyses

One-way analysis of variance (ANOVA) was performed to compare continuous variables. The Brown-Forsythe test was used when the data were not homoscedastic. In the cases of homogeneity of variance, we adopted the least significant difference (LSD) method for post-hoc testing. On the contrary, we used the Tamhane T2 test when the data were not homoscedastic. The Pearson χ^2^ test and Fisher exact test were used to compare categorical variables between the three groups. Pearson’s correlation was used in the correlation analysis of age, educational level, and cognitive scores. The correlation between Suzuki stages and cognitive scores was analyzed using Spearman’s rank correlation. Differences were considered statistically significant at a *P*-value< 0.05. Statistical analyses were performed using SPSS software, version 20.0 (IBM Corp, Armonk, NY).

## Results

A total of 40 patients met the inclusion criteria. We divided the patients into two subgroups based on the presence of symptoms and the area of cerebral infarction. There were 19 patients in the cerebral infarction group and 21 patients in the asymptomatic group. The mean ages of patients in the cerebral infarction group, asymptomatic patient group, and healthy control group were 41.89, 39.38, and 42.55 years, respectively. The characteristics of the three groups were relatively balanced in terms of sex, age, and educational background (Table 1). The clinical information of the patients is described in detail in Table 1. An ANOVA was conducted to evaluate the effect of the inclusion of clinical variables on cognition. Although there was a statistical difference in the history of hyperthyroidism between the three groups, the proportion of patients in the two patient groups was approximately the same.

**Table 1:** Characteristics and Clinical Information of Participants

	Infarction (*n* = 19) Mean ± SD	Asymptomatic (*n* = 21) Mean ± SD	HC (*n* = 20) Mean ± SD	*P*-value
Sex (M:F)	10:9	14:7	12:8	0.664
Age, y	41.89 ± 11.65	39.38 ± 10.23	42.55 ± 13.85	0.672
Education, y	9.84 ± 2.75	9.52 ± 3.53	11.10 ± 3.95	0.316
Medical history, n (%)				
Hypertension	4 (21.1)	5 (23.8)	5 (25)	0.182
Diabetes mellitus	2 (10.5)	1 (4.8)	2 (10)	0.994
Dyslipidemia	5 (26.3)	2 (9.5)	2 (10)	0.418
Smoking history	6 (31.6)	4 (19)	3 (15)	0.177
Alcohol taking	3 (15.8)	2 (9.5)	2 (10)	0.510
Hyperthyroidism	1 (5.3)	1 (4.8)	0 (0)	0.000
Suzuki Stage				
Left				
No findings	1 (5.3)	1 (4.8)		
1	1 (5.3)	1 (4.8)		
2	3 (15.8)	3 (14.3)		
3	7 (36.8)	13 (61.9)		
4	4 (21.1)	1 (4.8)		
5	3 (15.8)	1 (4.8)		
6	0	1 (4.8)		
Right				
No findings	1 (5.3)	1 (4.8)		
1	0	3 (14.3)		
2	5 (26.3)	4 (19.0)		
3	9 (47.4)	9 (42.9)		
4	1 (5.3)	2 (9.5)		
5	3 (15.8)	1 (4.8)		
6	0	1 (4.8)		

Abbreviations: *F* Female, *M* Male, *SD* Standard deviation

Values are numbers of cases (%) unless otherwise indicated. Mean values are presented with SDs.

The Initial symptom of patients in the cerebral infarction group were 11 TIA, 7 headache and 1 epilepsy. MRS score in the cerebral infarction group was 1 in fifteen patients and 2 in four patients. The disease duration was 21.26 ± 23.54 and 16.48 ± 20.78 in the cerebral infarction group and the asymptomatic group, respectively.

In the asymptomatic subgroup, 9 people (42.8%) were normal in the Fazekas rating of white matter, 10 were in grade one (47.6.2%), and 2 were in grade two (9.5%). And there were 4 patients (19.0%) with no obvious infarction lesion, 14 patients (66.6%) with 1–3 silent cerebral infarction lesions with diameter of 0.5-15 mm, and 3 patients (14.2%) with 3 or more lesions. Most silent cerebral infarction lesions are located in the parietal lobe of the brain.

### Differences in cognitive dysfunction among patients with different clinical subtypes

**Fig. 1:**
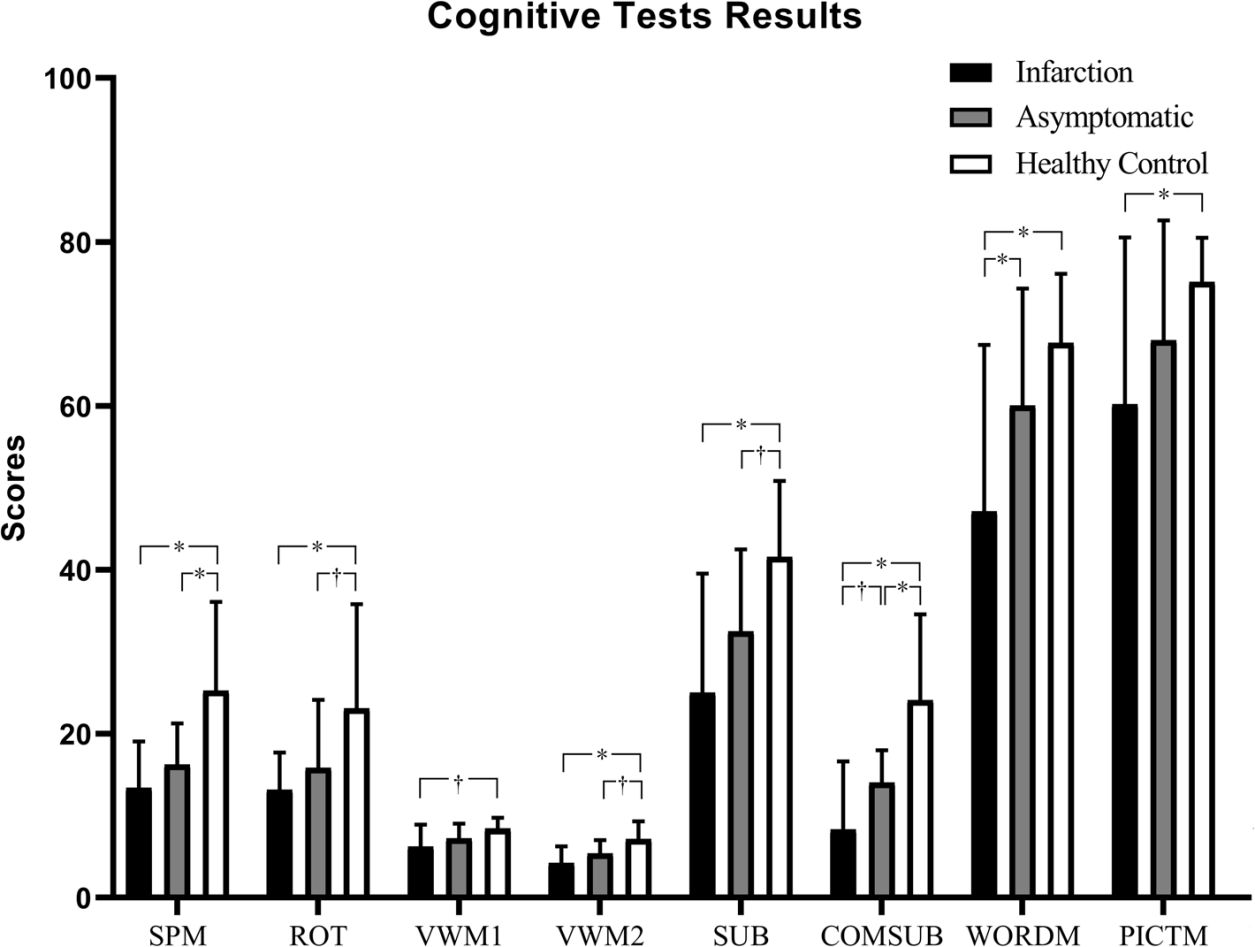
Differences in cognitive dysfunction among patients with different clinical subtypes. *: ≤0.01. †: < 0.05 and > 0.01. Abbreviations: Raven’s Standard Progressive Matrices; ROT, mental rotation; VWM1, verbal working memory-forward digit span task, VWM2, verbal working memory-backward digit span task; SUB, simple subtraction; COMSUB, complex subtraction; WORDM, word-memory; PICTM, picture-memory

As shown in Table 2, we used an ANOVA to analyze the data from the three groups and significant statistical differences among the three groups for the results of Raven’s Standard Progressive Matrices, three-dimensional rotation, verbal working memory, computational ability, and short-term memory. The results of the post-hoc tests in the three groups are presented in Fig. 1.

**Table 2:** Neuropsychological assessments

	Infarction (*n* = 19)	Asymptomatic (*n* = 21)	HC (*n* = 20)	*p* value
SPM	13.37 ± 5.66	16.48 ± 5.07	25.25 ± 10.86	0.000
ROT	13.11 ± 4.58	15.81 ± 8.33	23.1 ± 12.74	0.004
VWM1	6.21 ± 2.68	7.24 ± 1.76	8.40 ± 1.31	0.006
VWM2	4.21 ± 2.04	5.39 ± 1.65	7.15 ± 2.13	0.000
SUB	25.00 ± 14.51	32.48 ± 10.00	41.55 ± 9.31	0.000
COMSUB	8.26 ± 8.33	14.00 ± 3.95	24.10 ± 10.49	0.000
WORDM	47.11 ± 20.83	60.05 ± 14.26	67.65 ± 8.46	0.000
PICTM	60.21 ± 20.33	68.00 ± 14.65	75.10 ± 5.41	0.010

Abbreviations: *AS* Asymptomatic, *SD* Standard deviation, *SPM* Raven’s Standard Progressive Matrices, *ROT* Mental rotation, *VWM1* Verbal working memory-forward digit span task, *VWM2* Verbal working memory-backward digit span task, *SUB* Simple subtraction, *COMSUB* Complex subtraction, *WORDM* word-memory, *PICTM* Picture-memory

Compared with the healthy control group, the patients with cerebral infarction had significant impairments of intelligence, spatial imagination, working memory, computational ability, and short-term memory. In the forward digit span task, although there was a statistical difference, the degree of decline was not as obvious as that in the other tests (VWM1, *P* = 0.01).

Compared with the healthy control group, asymptomatic patients showed varying degrees of decline in intelligence (SPM, *P* = 0.001), spatial imagination (ROT, *P* = 0.014), working memory (VWM2, *P* = 0.011), and computational ability (SUB, *P* = 0.014; COMSUB, *P* < 0.001).

The decline in complex arithmetic ability (COMSUB, *P* = 0.027) and short-term memory of words (WORDM, *P* = 0.01) was more severe in patients with cerebral infarction than that in the asymptomatic group.

### The correlation between age, education level, and cognition

SPM scores of patients with cerebral infarction negatively correlated with age (*r* = − 0.376, *p* = 0.04), and the backward digit span task result negatively correlated with age (*r* = − 0.566, *p* = 0.011). In the asymptomatic group, mental rotation results positively correlated with educational level (*r* = 0.589, *p* = 0.005) and the forward digit span task results positively correlated with educational level (*r* = 0.543, *p* = 0.011). There was no significant correlation between suzuki stage and other clinical underlying diseases and scores of cognitive scale.

In the analysis of the results of the healthy control, the negative correlation between SPM and age also appeared (*r* = − 0.396, *p* = 0.026). Short-term memory also showed a negative correlation with age (*r* = − 0.491, *p* = 0.028). There was a positive correlation between scores of complex arithmetic and years of education (*r* = 0.586, *p* = 0.007).

## Discussion

Previous studies have shown cognitive impairment in adults with MMD. However, the role of stroke in this cognitive decline and when cognitive impairment occurs remains unclear. The current study is the first to describe cognitive effects in a large group of adults with asymptomatic MMD and those with MMD with a history of cerebral infarction. The current findings suggest that patients with MMD with a history of cerebral infarction were significantly impaired in all of the tested cognitive domains, but that patients with cerebral infarction were only more severely affected in complex number operations and short-term memory of words than asymptomatic patients. Patients with asymptomatic MMD have experienced significant cognitive decline prior to the onset of clinical symptoms.

### MMD and mathematical performance

The literature has shown that the ability to perform complex number operations is often associated with the parietal and frontal lobes [28]. A functional near-infrared spectroscopy (NIRS) study has suggested that the frontal lobe is involved in complex mathematical calculations, while the left frontal lobe is activated by detailed processing and the right frontal lobe is activated by holistic processing [29]. In our population of patients with cerebral infarction, 11 had infarcts in the frontal lobe, accounting for approximately 57.9% of patients. The cognitive results are highly consistent with the damaged brain area. The main function of the temporal lobe is not fully understood, but auditory perception is one of its main functions. Some visual skills and language, memory, and some motor functions may depend on the temporal lobe [30, 31].

In the cerebral infarction group, 15 patients (78.9%) had independent or combined lesions of the temporal lobe. The understanding of Chinese characters is relatively different from reading in other languages; therefore, the short-term memory of Chinese words is easily affected by education level and other similar factors. The results of one of these studies suggest that the temporal lobe is the central region for processing word information and is important for word understanding [32]. This may be related to the decline in the infarction group’s short-term verbal memory. However, studies on the formation of language and activation of various brain regions are not exact, even in the special language form of Chinese [33]. Specific analyses need to be performed using cognitive and functional magnetic resonance imaging (fMRI) data.

### MMD and STM performance

In the post-hoc analysis of cerebral infarction and asymptomatic patients, the short-term memory of the images exhibited negative results. We may find that some images are easier to remember than words, and similarly, education level has less effect on short-term memory of images than that of words. Studies have also shown that memory declines are less pronounced over time [34].^,^ [35] In addition, the backward digit span task is related to execution function and the reduction of this index is consistent with previous research results [36].

### Correlational analysis of age, education degree, and cognition

In the healthy control group, complex arithmetic performance positively correlated with years of education (*r* = 0.586, *p* = 0.007), while intelligence and short-term memory of words negatively correlated with age. Moreover, the cognitive performance of patients showed that education level was an important positive factor affecting cognitive function, while the age factor was more negative. This phenomenon is consistent with other cognitive research conclusions [37].

### Cognitive performance of different subgroups

Patients with cerebral infarction were cognitively impaired and performed poorly in all tests. In combination with their clinical symptoms, active treatment, such as revascularization, should be given priority. Results of previous studies have shown that asymptomatic MMD is not a silent disease and may cause cerebrovascular accidents, such as ischemic or hemorrhagic strokes [12]. Our findings suggest that asymptomatic MMD patients experience cognitive impairment in areas such as intelligence, spatial ability, verbal working memory, and number manipulation, suggesting that cognitive impairment is a long-term complication of conservative treatment of asymptomatic MMD. Compared with asymptomatic patients, patients with cerebral infarction had more severe cognitive impairment only in terms of complex subtraction and short-term memory.

In a study of the effect of secondary brain damage of MMD on cognition, Mogensen et al. performed executive function tests on 31 stroke patients and found that secondary damage such as cortical stroke and white matter damage were associated with decreased executive function. The executive function of adult MMD decreases significantly with cortical stroke and white matter lesions in the posterior (parietal occipital region). The cognitive results of this study were consistent with our subgroup of infarction, which performed worse on tests of verbal working memory related to executive function. It should be noted that in this study, asymptomatic moyamoya patients also had decreased executive ability compared with the normal control group (VWM2, *P* = 0.011), while in asymptomatic moyamoya patients included in this study, 14 patients (61%) had cerebral white matter abnormalities. And a total of 15 people (65.2%) with silent brain infarctions with a diameter of 0.5–15 mm had 1–3 lesions, and 4 patients (17.4%) had more than 3 lesions. Therefore, the appearance of cognitive impairment in patients with asymptomatic moyamoya disease may be related to silent cerebral infarction and secondary white matter lesions, and changes in cognitive abilities of lesions at different anatomical locations need further study [38].

A meta-analysis of 17 studies of cognitive impairment in children and adults with MOyamoya disease suggested that the median percentage of cognitive impairment in adults was 31% (range 0 to 69%), with multiple domains affected. The proportionally inconsistent impairments observed in different cognitive domains in adults may be due to different inclusion criteria and testing methods in the studies included. The study also suggests that it is not clear whether MMD directly affects cognition through chronic hypoperfusion or whether cognitive impairment is primarily the result of stroke. In our study included in asymptomatic patients with moyamoya disease was observed, and no factors of stroke patients are different degree of cognitive impairment, the current can be set up such a hypothesis, their cerebral hemodynamic damaged or patients with moyamoya disease is the main cause of cognitive impairment, cerebral infarction and other secondary damage is an important contributing factor of cognitive impairment [15].

However, the asymptomatic stage of MMD is relatively early. A clear pattern was found in the post-hoc examination that there was no significant difference in most cognitive measures between the cerebral infarction and asymptomatic group, while there was a significant difference between the asymptomatic group and normal control group. Clinical symptoms may worsen after a cerebral infarction or other cerebrovascular accident; however, advanced cognitive function is impaired more severely before symptoms appear. The occurrence of cerebral infarction may result in specific cognitive impairment due to damage to specific functional areas of the brain. Therefore, we found a mismatch between symptoms and cognitive impairment. Our findings suggest that doctors treating MMD patients should pay attention to not only to clinical cerebrovascular accidents but also cognitive decline and comprehensively judge the timing of treatment.

### Strengths and limitations

Our study found that patients with MMD had varying degrees of cognitive impairment prior to the onset of clinical symptoms. However, longitudinal studies are still needed to identify natural changes in neurocognitive function in conservatively treated patients. However, in a previous cognitive study, use of the Montreal Cognitive Assessment in patients with hemorrhagic MMD revealed mild cognitive impairment at follow-up. This may indicate that patients with hemorrhagic MMD have more severe cognitive impairment due to cerebrovascular accidents [39]. We will include a bleeding subgroup for comparison in the follow-up study. Moreover, due to limitations of sample size, it was hard to conduct a clinical subtype analysis in patients with ischemia. In addition, we will describe the cognitive outcomes of different treatments, such as conservative treatment and revascularization.

## Conclusions

Our study found a mismatch between the clinical symptoms and cognitive impairment in patients with MMD. People s with asymptomatic MMD have cognitive impairments in intelligence, spatial ability, verbal working memory, and number manipulation. Compared with asymptomatic patients, patients with cerebral infarction had more severe cognitive impairment only in terms of complex subtraction and short-term memory. Therefore, cognitive impairment may be a long-term complication of conservative treatment in asymptomatic MMD patients.

## Data Availability

All data generated or analysed during this study are included in this published article. Some or all data, models, or code generated or used during the study are available from the corresponding author by request.

## Abbreviations

MMD: Moyamoya disease
MRI: Magnetic Resonance Imaging
MRA: Magnetic Resonance Angiography
SD: Standard deviation
HC: Healthy control
